# Plasma levels of interleukin 27 in falciparum malaria is increased independently of co-infection with HIV: potential immune-regulatory role during malaria

**DOI:** 10.1186/s12879-020-4783-8

**Published:** 2020-01-21

**Authors:** Kari Otterdal, Aase Berg, Annika E. Michelsen, Sam Patel, Ida Gregersen, Ellen Lund Sagen, Bente Halvorsen, Arne Yndestad, Thor Ueland, Nina Langeland, Pål Aukrust

**Affiliations:** 1 Research Institute of Internal Medicine, Oslo University Hospital Rikshospitalet, PO Box 4950, 0424 Oslo, Nydalen Norway; 2 Department of Medicine, Stavanger University Hospital, PO Box 8100, 4068 Stavanger, Norway; 3 Department of Medicine, Central Hospital of Maputo, 1100 Maputo, Mozambique; 4 Faculty of Medicine, University of Oslo, 0316 Oslo, Norway; 5 K.G. Jebsen Inflammatory Research Center, University of Oslo, 0424 Oslo, Norway; 6 K.G. Jebsen Thrombosis Research and Expertise Center, University of Tromsø, 9019 Tromsø, Norway; 7 Department of Clinical Science, University of Bergen, 5021 Bergen, Norway; 8 Department of Medicine, Haukeland University Hospital, 5021 Bergen, Norway; 9 Department of Medicine, Haraldsplass Deaconess Hospital, 5009 Bergen, Norway; 10 Section of Clinical Immunology and Infectious Diseases, Oslo University Hospital Rikshospitalet, 0372 Oslo, Norway

**Keywords:** Falciparum malaria, HIV, IL-27, Endothelial cells, PBMC, Hemozoin, Interleukins

## Abstract

**Background:**

The immune response during falciparum malaria mediates both harmful and protective effects on the host; however the participating molecules have not been fully defined. Interleukin (IL)-27 is a pleiotropic cytokine exerting both inflammatory and anti-inflammatory effects, but data on IL-27 in malaria patients are scarce.

**Methods:**

Clinical data and blood samples were collected from adults in Mozambique with *P. falciparum* infection, with (*n* = 70) and without (*n* = 61) HIV-1 co-infection, from HIV-infected patients with similar symptoms without malaria (*n* = 58) and from healthy controls (*n* = 52). In vitro studies were performed in endothelial cells and PBMC using hemozoin crystals. Samples were analyzed using enzyme immunoassays and quantitative PCR.

**Results:**

(i) IL-27 was markedly up-regulated in malaria patients compared with controls and HIV-infected patients without malaria, showing no relation to HIV co-infection. (ii) IL-27 was correlated with *P. falciparum* parasitemia and von Willebrand factor as a marker of endothelial activation, but not with disease severity. (iii) In vitro, IL-27 modulated the hemozoin-mediated cytokine response in endothelial cells and PBMC with enhancing effects on IL-6 and attenuating effects on IL-8.

**Conclusion:**

Our findings show that IL-27 is regulated during falciparum malaria, mediating both inflammatory and anti-inflammatory effects, potentially playing an immune-regulatory role during falciparum malaria.

## Background

Infection with *Plasmodium falciparum* (*P. falciparum*) is associated with a marked increase in systemic and local inflammation, potentially contributing to the pathogenesis of malaria rather than being protective [1–3]. However, the immune response during *P. falciparum* infection is rather complex, consisting of both adaptive and maladaptive signaling [4]. Falciparum malaria infection triggers a broad range of cytokines [Interleukin (IL)-1ra, IL-6, IL-8, IL-9, IL-10, Eotaxin, Interferon gamma-induced protein 10 (IP-10), monocyte chemotactic protein-1 (MCP-1), macrophage inflammatory protein-1β (MIP-1β) and tumor necrosis factor (TNF)]. Of those, TNF, IL-8 and IP-10 are associated with increased severity and IL-8 and Eotaxin with malaria and HIV co-infection [5, 6]. Thus, in addition to characterizing activation of inflammatory pathways that contribute to disease severity, it is of major importance to identify mediators that could mediate protective responses for the host. Hence, whereas TNF is regarded as a prototypical inflammatory mediator during falciparum malaria promoting organ failure and disease severity [6], the anti-inflammatory cytokine IL-10 may be of importance in preventing T cell- and cytokine-mediated pathology during potentially lethal malaria infections [7]. However, an overwhelming anti-inflammatory response may also be harmful for the host, and the identification of protective and harmful mediators and the balance between these molecules during falciparum malaria is far from clear.

IL-27 is a pleiotropic two-chain cytokine, composed of EBI3 (Epstein-Barr virus-induced gene 3) and IL-27p28 subunits related to both the IL-12 and IL-6 cytokine families. IL-27 may exert both inflammatory and anti-inflammatory effects in a context dependent manner, partly determined by disease category and state [8–10]. In experimental malaria, IL-27 has been suggested to regulate protective immunity partly through IL-27 producing CD4^+^ T cells [11]. However, data on IL-27 regulation in clinical malaria is scarce, and to this end, there are no data on IL-27 levels during falciparum malaria in adults. Further, how co-infection with HIV influences IL-27 levels during falciparum malaria is unknown and such knowledge would be of importance in light of a considerable geographic overlap between the two diseases, particularly in sub-Saharan Africa where different interactions between HIV and malaria has been described [12, 13].

To examine the role of IL-27 in falciparum malaria, plasma IL-27 was measured in a cohort of adult patients with *P. falciparum* infection and related to disease severity and parasitemia as assessed by quantitative *P. falciparum* PCR analyses. The study was performed in Mozambique which has one of the highest global incidences of co-infection with HIV and falciparum malaria. We therefore also examined the association between HIV infection and IL-27 levels. Finally, to elucidate any potential consequences of altered IL-27 levels during falciparum malaria in vivo, we examined the ability of IL-27 to modulate hemozoin-induced release of various inflammatory cytokines in peripheral blood mononuclear cells (PBMC) and endothelial cells.

## Methods

### Description of study design and participants

The study design has previously been described [12]. Briefly, during 7 months in two malaria peak seasons, from 2011 to 2012 we included all patients (*n* = 212) admitted to the Medical Emergency Department in the Central Hospital of Maputo, Mozambique. The inclusion criteria in this prospective, cross-sectional study, were age ≥ 18 years, non-pregnancy, axillary temperature ≥ 38 °C and/or clinical suspected or confirmed malaria infection, and consent from patient or next of kin. Clinical suspicion of malaria was defined as a history of fever, chills, headache, mental confusion, dyspnea, vomiting and/or diarrhea, myalgia and/or general malaise in the absence of other symptoms and findings indicating other severe infections or conditions. Pregnancy was an exclusion criteria due to the different immune response compared to non-pregnancy [14, 15]. Of the 212 screened patients, 129 had *P. falciparum* malaria as assessed by qualitative PCR and two had rapid diagnostic test (RDT) and malaria slide positive for *P. falciparum* giving a total of 131 malaria patients (median age 37 years [18–84 years], 47% women, 53% co-infected with HIV-1 [PCR and/or serological tests]). Of the malaria patients, 92% received quinine intravenously, 4% received artemether intramuscularly, and the rest were treated with oral artemisinin combinations [12].

Severe malaria was defined according to WHO definitions [16]. Severe malaria was found in 65% (85/131) of the patients and 13% (17/131) had very severe malaria defined as three or more severity criteria [12]. Of the malaria patients 7.6% died (10/128 of which 9 were co-infected with HIV; missing data on outcome in 3 patients). The characteristics of the patient groups at admission are shown in Table 1, including data on CD4 T cell counts, plasma levels of HIV RNA and antiretroviral treatment (ART). The qualitative *P. falciparum* PCR in whole blood were performed as previously described [17, 18]. Estimated glomerulus filtration rate (eGFR) was calculated from the abbreviated MDRD (Modification of Diet in Renal Disease) equation based on measured serum creatinine, age, sex and race.

**Table 1:** Clinical characteristics^a)^ of the patient population at admission^b)^

	HIV only	Malaria only	Malaria and HIV
N	58	61	70
Age, years	39 (22–84)	40 (18–79)	40 (20–65)
Sex, females (%)	50 (29/58)	41 (25/61)	50 (35/70)
Hemoglobin (g/dL)	8.9 (2.9–15.2)	11.2 (3.2–17.0)	9.4 (2.5–15.7)
Leukocytes (× 10^9^/L)	8.2 (0.3–25.4)	6.9 (1.3–15.5)	7.8 (0.9–21.8)
Platelets (×10^9^/L)	220 (13–682)	124 (11–452)	90 (8–330)
Se-Creatinine (μmol/L)	161 (41–873)	127 (57–357)	223 (62–1529)
Se-Glucose (mmol/L)	6.1 (3.3–10.6)	8.7 (3.6–40.5)	6.12 (1.5–27.0)
Liver failure (%)^c)^	5 (4/57)	5 (3/61)	17 (12/70)
Coagulation disturb. (%)^d)^	0	2 (1/61)	13 (9/70)
Cerebral affection (%) ^e)^	33 (19/58)	25 (15/61)	31 (22/70)
Systolic blood pressure	115 (90–160)	122 (70–240)	115 (80–170)
Respiratory rate	29 (12–56)	22 (12–68)	24 (16–42)
CD4 T-cells (10^6^/l)^f)^	120 (10–196)	0	221 (14–632)
HIV-RNA (copies/ml)	2.6 × 10^4^ (0–5.1 × 10^5^)	0	4.2 × 10^4^ (0–8.3 × 10^5^)
ART before admission	29 (17/58)	0	19 (13/70)
Effective ART^g)^	17 (10/58)	0	13 (9/70)
Case fatality rate (%)	27.8 (15/54)	1.7 (1/59)	13.0^h)^ (9/69)

^a)^ Values in mean (min-max) or percentage and proportion. ^b)^ The 52 healthy controls are not included. ^c)^ Defined as jaundice/ bilirubine> 50 μmol/L ^d)^ Defined as bleeding disturbances/ hemolysis ^e)^ Defined as GCS ≤ 11, convulsions or confusion ^f)^CD4 T-cell count were only obtained in 8 (HIV only) and 11 (HIV + malaria) patients. ^g)^ Effective ART is defined as undetectable HIV-RNA levels. ^h)^ One patient died of non-malarial cause, he was excluded

For comparison, we also included 58 HIV-1-infected patients, admitted with clinical suspicion of malaria (i.e., similar symptoms) as mentioned above, but where malaria was excluded. These patients were diagnosed with among others tuberculosis, bacterial pneumonia, viral hepatitis, *Pneumocystis jirovecii* pneumonia, toxoplasma encephalitis, urinary tract infection and sepsis. Fifty-two apparently healthy HIV negative and malaria negative volunteers with median age 29 years (18–56 years), and 40% women, were enrolled from hospital employees provided no history of chronic disease, a subjective feeling of wellbeing and a healthy appearance evaluated by the researchers.

### Blood sampling protocol

Blood samples from patients and healthy controls were collected from peripheral vein into pyrogenic-free EDTA-tubes that were immediately placed on ice, and centrifuged within 30 min at 2000 *g* for 20 min to obtain platelet poor plasma. Plasma was thereafter aliquoted and stored at -80 °C. Sample 1 was done on admission and sample 2 after 48 h.

### The quantitative *P. falciparum* PCR in plasma

The concentration of *P. falciparum* DNA in plasma was measured by real-time quantitative PCR (qPCR) as previously described [17, 19]. Briefly, samples were run on LightCycler® 480 Multiwell Plate 384, white (Roche Diagnostics, Mannheim, Germany) using Primer Pf-1 (5′-ATT GCT TTT GAG AGG TTT TGT TAC TTT-3′), primer Pf-2 (5′-GCT GTA GTA TTC AAA CAC AAT GAA CTC AA-3′) and probe Pf (5′-CAT AAC AGA CGG GTA GTC AT-3′) (Applied Biosystems, Cheshire, UK). DNA quantity for samples with *P. falciparum* DNA less than the Limit of Quantification (LOQ) was set to be equal to or less than the LOQ (estimated to ≤6.4 parasites/μl).

### Isolation and culturing of PBMC

To obtain PBMC, heparinized blood from healthy controls was subjected to Isopaque-Ficoll gradient centrifugation and seeded in 48-well trays (10^6^/mL; Thermo Scientific) in RPMI 1640 (PAA Laboratories, Pasching, Austria) supplemented with 10% fetal bovine serum (FBS; Gibco, Grand Island, NY) as previously described [20]. The cells were cultured with recombinant human (rh)IL-27 (100 ng/mL; R&D Systems, Minneapolis, MN) in RPMI 1640 supplemented with 10% FBS for 1 h before stimulated with different concentrations of chemically synthesized hemozoin (Invivogen, San Diego, CA) for 22 h.

### Endothelial cell culture

Primary Human Aortic Endothelial cells (HAoECs) were obtained from PromoCell GmbH, Heidelberg, Germany. The cells were cultured in Endothelial Cell Growth Medium MV2 (PromoCell), passaged by treatment with Trypsin/EDTA (0.04%/0.03%; PromoCell) and grown in 48-well plates (Thermo Scientific, Roskilde, Denmark) coated with 1% gelatin (Sigma, St Louis, MO). The cells were plated one or two days before experimental start aiming 90% confluence. The cells were stimulated in the manner as described for PBMC using Opti-MEM reduced serum medium (Gibco) supplemented with 5% FBS. For evaluation of possible cell toxicity, different concentration of hemozoin was tested in both HAoEC and PBMC cultures where lactate dehydrogenase was quantified in fresh cell supernatants using Cytotoxicity Detection Kit from Sigma Aldrich (St. Louis, MO). In the HAoEC cultures cytotoxicity was observed with the highest hemozoin concentration tested (200 μg/mL) and this hemozoin concentration was therefore excluded in further experiments with endothelial cells.

### Supernatant and plasma analyses

Plasma levels of IL-27 and IL-6 and IL-8 levels in cell supernatants were measured by enzyme immunoassays (EIAs) from R&D Systems. von Willebrand factor (vWF) levels in plasma were measured by EIA with antibodies from Dako Cytomation (Glostrup, Denmark). The intra- and interassay coefficient of variation were < 10% for all assays.

### Real-time quantitative RT-PCR for in vitro samples

Total RNA was obtained from HAoEC and PBMC and real-time qPCR analyses were performed as previously described [20]. mRNA detection of gp130 and reference genes GAPDH and β-actin was assessed with SybrGreen primers (Sigma Aldrich, St. Louis, MO 63103): gp130, forward primers (FP): CATCGCACCTATTTAAGAGGGAACT, reverse primers (RP): CCTTTGGAAGGTGGAGCTTGT; GAPDH, FP: GCCCCCGGTTTCTATAAATTG, RP: GTCGAACAGGAGGAGCAGAGA; β-actin, FP: AGGCACCAGGGCGTGAT, RP: TCGTCCCAGTTGGTGACGAT. Sequence specific TaqMan primers and probes were used for detection of IL-27Rα mRNA (Assay-ID: Hs00945029_m1; Applied Biosystems). The relative mRNA level of each transcript was calculated by the ΔΔCt-method and normalized to controls.

### Statistical analyses

The distribution of inflammatory markers was skewed and nonparametric statistics were used throughout. For comparison between the diagnostic groups, Kruskal-Wallis was used a priori followed by Dunn’s multiple comparison test between individual groups. Wilcoxon matched-pairs signed rank test was used to compare changes from baseline to follow-up within each diagnostic group. Comparison of IL-27 in patients with and without severe malaria was performed using the Mann-Whitney U-test. Spearman correlation was used to assess associations between variables. In the ex vivo experiments Student’s t test was used. A two-sided *p* < 0.05 was considered significant.

## Results

### IL-27 in *P. falciparum* infection with and without HIV infection

**Fig. 1:**
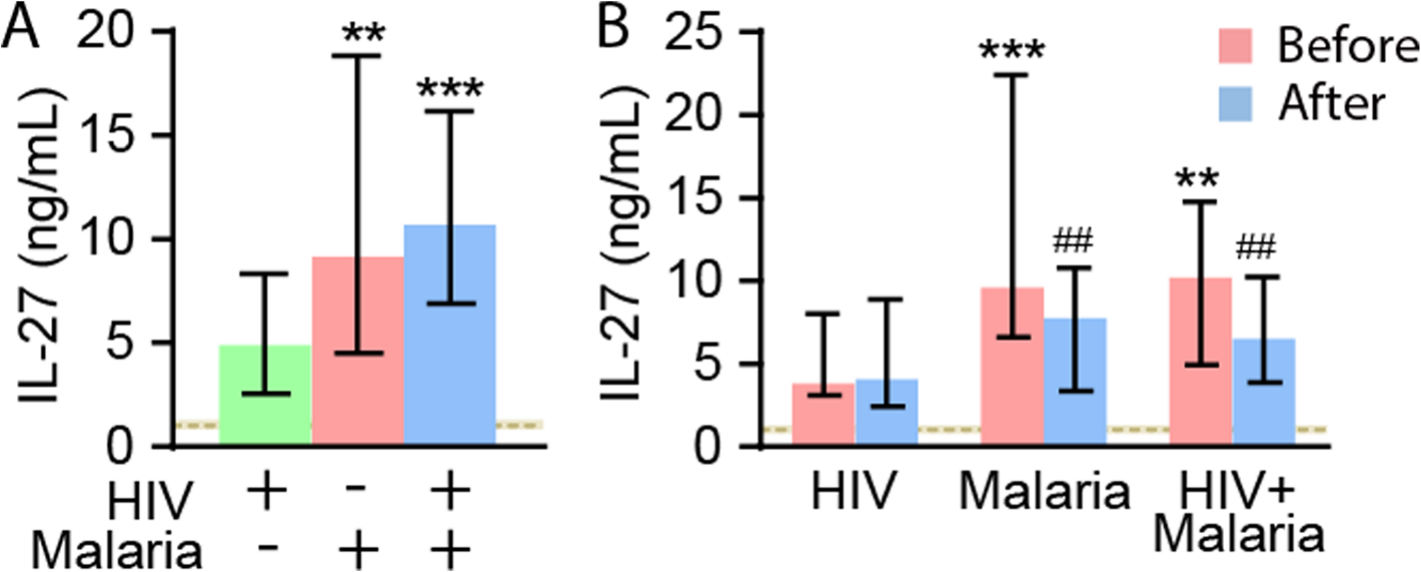
Plasma levels of IL-27 in the patient groups. **a** shows plasma levels of IL-27 in patients with HIV infection with febrile symptoms but without malaria (*n* = 58), patients with falciparum malaria without (*n* = 61) and with HIV infection (*n* = 70). **b** shows plasma levels of IL-27 during baseline and follow-up that were available in 49 patients with HIV infection without malaria and in patients with falciparum malaria without (*n* = 6) and with HIV infection (*n* = 22) at admission (before) and 48 h thereafter (after). Data are given as median and 25-75th percentiles. **p < 0.01 and ***p < 0.001 versus HIV without malaria. ##*p* < 0.01 versus levels at admission. The horizontal dashed line and shaded area represent median levels and 25-75th percentiles in healthy controls (*n* = 52). IL-27 levels were significantly raised compared with levels in controls in all three groups of patients (*p* < 0.001 for all comparisons)

As can be seen in Fig. 1a, IL-27 was significantly increased in both the malaria groups as compared with healthy controls and HIV-infected patients with similar febrile symptoms, but without malaria. There were no differences between patients with falciparum malaria with and without co-infection with HIV, indicating that the elevated IL-27 levels are mainly associated with malaria. In the malaria patients as a whole, IL-27 levels were negatively correlated with platelets count independently of co-infection with HIV, indicating an association with platelet activation (Table 2). In malaria patients IL-27 levels were also negatively correlated with eGFR, reaching statistical significance in those co-infected with HIV. In contrast, there was no correlation between IL-27 and leukocyte counts, lymphocyte counts or granulocyte counts with the same pattern in the two malaria groups (Table 2).

**Table 2:** Correlation between IL-27 and clinical data in malaria patients with (*n* = 70) and without (*n* = 61) HIV and in HIV infected patients without malaria (*n* = 58)

	Malaria		Malaria+HIV		HIV only	
	n	*r*	n	*r*	n	*r*
qMalPCR	60	0.63**	67	0.62**	–	–
eGFR	45	−0.27	60	−0.29*	43	−0.20
Platelets	53	−0.47**	63	−0.36**	47	−0.46**
Neutrophils	42	0.15	43	− 0.08	39	0.39*
Lymphocytes	32	−0.21	32	−0.06	20	−0.37
WBC	53	0.08	64	0.07	48	−0.16

Not all data were available in all patients. *eGFR* estimated glomerular filtration rate, *qMalPCR* quantitative PCR of falciparum malaria in plasma; severity, disease severity according to WHO classification, *WBC* White blood cell counts. *Correlation is significant at the 0.05 level (2-tailed). **Correlation is significant at the 0.01 level (2-tailed)

### IL-27 in relation to degree of parasitemia, clinical disease severity and endothelial cell activation

In 93 of the 131 malaria patients, the degree of malaria parasitemia could be assessed by qPCR (38 patients had plasma levels below the detection limit of the assay). As shown in Table 2, IL-27 was strongly correlated with the degree of parasitemia with the same pattern in those with and those without co-infection with HIV. In contrast, IL-27 was not associated with disease severity as assessed by the WHO definition [16] in either of the two malaria groups. Thus, no differences within the malaria group (without vs severe): median 8.2 [25th 3.8, 75th 16.9] ng/mL vs. 9.9 [4.8, 26.1] *p* = 0.66 were observed and no differences were found within the HIV + malaria group (without vs severe): 12.6 [9.0, 15.9] vs. 9.6 [6.8, 16.2] *p* = 0.29. In the malaria group as a whole, no differences with regard to severity were observed (without vs with): 10.7 [5.1, 16.4] vs. 9.7 [5.9, 17.0] *p* = 0.90.

**Fig. 2:**
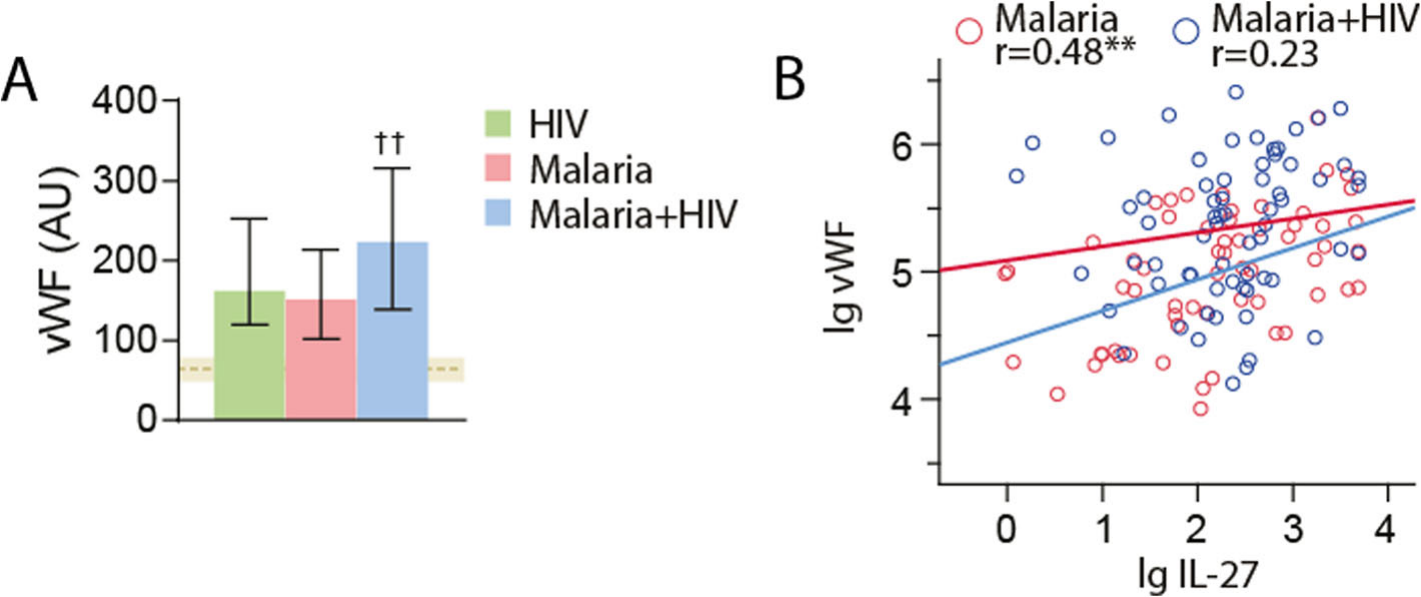
Plasma levels of von Willebrand factor (vWF) in the patient groups at admission. **a** shows plasma levels of vWF in patients with HIV infection with febrile symptoms but without malaria (*n* = 58), patients with falciparum malaria without (*n* = 61) and with HIV infection (*n* = 70). Data are given as median and 25-75th percentiles. ^††^*p* < 0.01 versus HIV without malaria and malaria without HIV. The horizontal dashed line and shaded area represent median levels and 25-75th percentiles in healthy controls (*n* = 52). vWF levels were significantly raised compared with levels in controls in all three groups of patients (*p* < 0.001 for all comparisons). **b** shows the correlation between plasma levels of IL-27 and vWF in patients with falciparum malaria with (*n* = 70) and without (*n* = 61) co-infection with HIV

Falciparum malaria affects endothelial cells, and as shown in Fig. 2a, all three groups of patients (HIV only, malaria only and HIV + malaria) had increased levels of vWF, as a reliable marker of endothelial cell activation compared with healthy controls, with the highest levels in those with both infections (Fig. 2a). Interestingly, plasma levels of IL-27 were positively correlated with vWF in patients with malaria alone and in HIV-infected patients without malaria (*r* = 0.54, *p* < 0.001), but not in those that were co-infected with HIV and malaria (Fig. 2b), potentially indicating some interactions between HIV and falciparum malaria that affects the pattern of endothelial cell activation.

### IL-27 levels in relation to clinical presentation of patients with severe malaria

Whereas there were no association between IL-27 levels and cerebral malaria (Glascow Coma Score ≤ 11), renal dysfunction (serum creatinine > 265 μM) and pulmonary oedema, IL-27 levels were significantly higher in those with severe anemia (< 5 g/dl) as compared with those without this manifestation (Table 3). Importantly, however, the number of patients in each subgroup was low, and all these data must be interpreted with caution. Moreover, statistical analyses were not performed for clinical manifestations that were seen in ≤5 patients (severe hypoglycemia and liver failure).

**Table 3:** IL-27 levels in relation to clinical presentation of patients with severe malaria

	Without affection		With affection		p
	N		N		
Cerebral malaria	38	9.49 (5.92–16.15)	37	9.78 (5.53–18.57)	0.910
Renal dysfunction	47	9.10 (5.46–13.79)	18	14.65 (7.05–22.08)	0.182
Pulmonary oedema	53	10.09 (5.65–18.73)	22	9.09 (6.82–12.14)	0.534
Severe anemia	61	10.58 (7.48–20.05)	13	5.46 (3.81–8.61)	0.004

Cerebral malaria (Glascow Coma Score < 11), renal dysfunction (serum creatinine > 265 μM), severe anemia (< 5 g/dl). Data given as median (25th–75th)

### The association of plasma levels of IL-27 and other inflammatory markers

We have previously shown that interferon-γ-induced protein 10 (IP-10/CXCL10), IL-8, soluble CD25 (sCD25) and terminal complement complex (TCC) are related to disease severity in this cohort [5, 19, 21]. We therefore next examined the association of IL-27 with these inflammatory markers. Whereas IL-27 levels were correlated with TCC in patients with falciparum malaria with and without HIV, but not in HIV-infected patients without malaria, IL-27 were correlated with IL-8 only in the latter group and notably, IL-27 levels were significantly correlated with IP-10 and sCD25 in all the three subgroups of patients (malaria only, malaria+HIV and HIV only) (Table 4). Both IP-10 (effects on T cells) and sCD25 (released from T cells upon activation) are related to T cell function/activation and these data further link IL-27 to T cell pathology during falciparum malaria.

**Table 4:** The association of plasma levels of IL-27 and other inflammatory markers in malaria patients with (*n* = 67) and without (*n* = 60) HIV and in HIV only (*n* = 58)

	Malaria		Malaria + HIV		HIV only	
	r	p	r	p	r	p
IL-8	0.144	0.271	0.139	0.261	0.590	< 0.001
IP-10	0.577	< 0.001	0.515	< 0.001	0.624	< 0.001
TCC	0.366	0.004	0.317	0.009	0.072	0.592
sCD25	0.740	0.001	0.300	0.015	0.580	< 0.001

Data are given as r and *p*-values

### IL-27 levels during follow-up

In 77 patients (HIV without malaria [*n* = 49], malaria only [*n* = 6], malaria and HIV [*n* = 22]) we also had follow-up samples taken in hospital 48 h after admission (Fig. 1b). Whereas there was a significant decline in IL-27 levels after 48 h, levels were still significantly increased as compared with HIV-infected patients without malaria and healthy controls. Importantly, patients with HIV infection without co-infection with malaria show no significant changes in IL-27 levels during follow-up (Fig. 1b).

### Effects of IL-27 on cytokine release in hemozoin-exposed endothelial cells

**Fig. 3:**
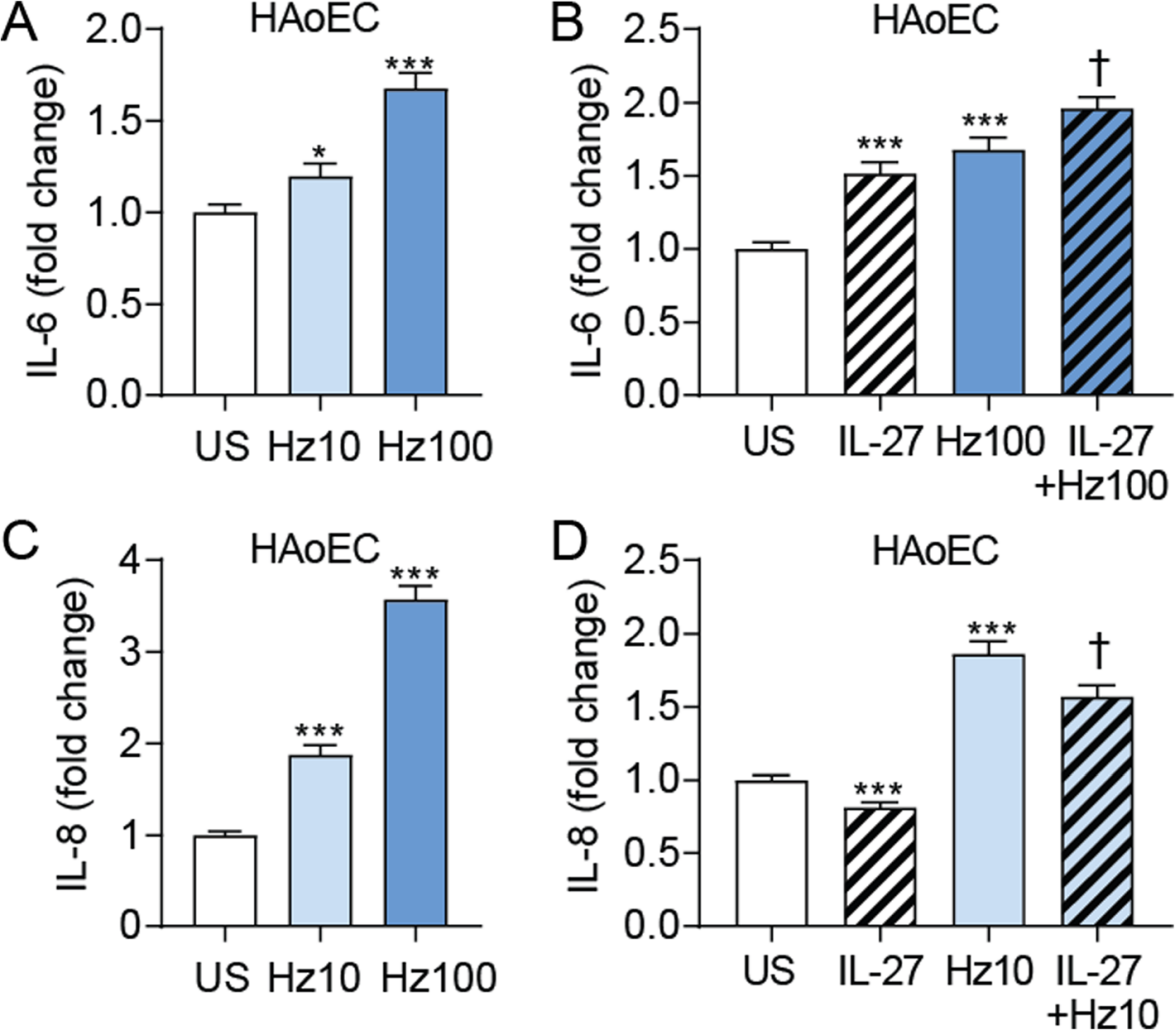
Effects of IL-27 on IL-6 and IL-8 release from hemozoin-exposed human aortic endothelial cells (HAoECs). Endothelial cells were primed with recombinant (rh)IL-27 (100 ng/mL, 90 min) and incubated with 10 and 100 μg/mL hemozoin (Hz) (indicated as Hz10 and Hz100) for 22 h. IL-6 (**a** and **b**) and IL-8 (**c** and **d**) was measured in supernatants from the cells with EIA. Data are presented as mean and SEM of four (IL-6 data) and five (IL-8 data) separate experiments and shown as fold change from control. *p < 0.05 and ***p < 0.001 versus unstimulated cells (US) (white bar), and †*p* < 0.05 versus Hz (blue bar)

Hemozoin is formed when plasmodium, during invasion of the red blood cells, digest hemoglobin [22]. To elucidate any possible consequences of the increased IL-27 levels in falciparum malaria, we examined the effect of IL-27 on the release of prototypical inflammatory cytokines (i.e., IL-6 and IL-8) in hemozoin-exposed HAoEC. Hemozoin caused a dose-dependent release of IL-6 that was further enhanced when co-incubated with rhIL27 (Fig. 3a-b). rhIL-27 also induced a release of IL-6 in unstimulated cells (Fig. 3b). Hemozoin also promoted a dose-dependent increase in IL-8 release, but in contrast to the effects on IL-6, rhIL-27 reduced the spontaneous and hemozoin-induced release of IL-8 from these cells (Fig. 3c-d). As seen in Fig. 3, the maximal effect of rhIL-27 in hemozoin-exposed cells was observed at different concentrations of hemozoin depending on the actual cytokine (i.e., 100 μg/mL for IL-6 and 10 μg/mL for IL-8), illustrating different sensitivity for IL-27-mediated modulation of hemozoin-effects on these cytokines.

### Effects of IL-27 on cytokine release in hemozoin-exposed PBMC

**Fig. 4:**
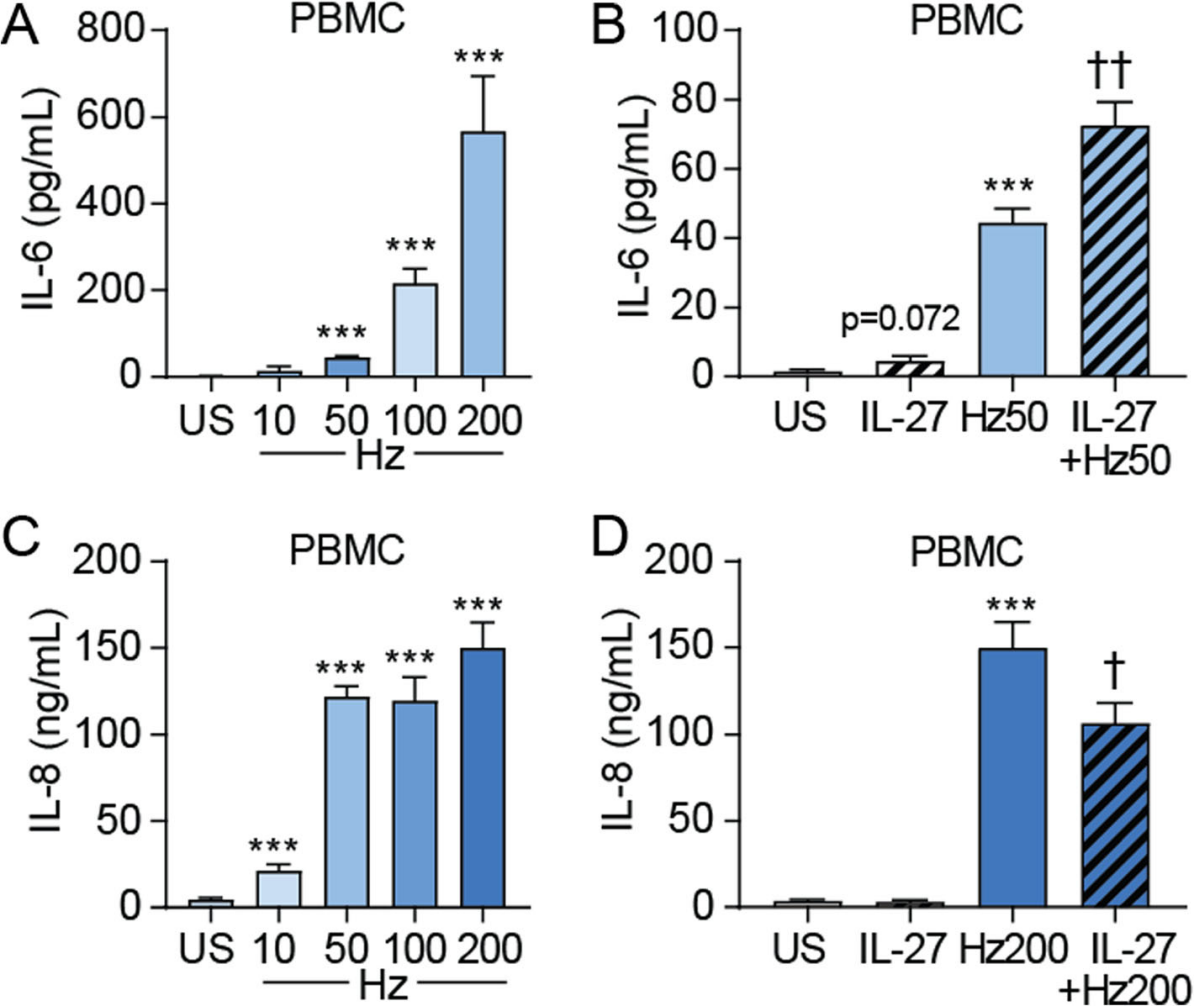
Effects of IL-27 on IL-6 and IL-8 release from hemozoin-exposed peripheral blood mononuclear cells (PBMCs). PBMCs were primed with recombinant human (rh)IL-27 (100 ng/mL, 90 min) and incubated with different concentrations of hemozoin (Hz) ranging from 10 to 200 μg/mL (indicated as Hz10, Hz50, Hz100 and Hz200) for 22 h. IL-6 (**a** and **b**) and IL-8 (**c** and **d**) was measured in supernatants from the cells with EIA. Data are presented as mean and SEM of three (IL-6 data) and five (IL-8 data) separate experiments. ***p < 0.001 versus unstimulated (US) cells (white bar), and †*p* < 0.05 and ††*p* < 0.01 versus Hz (blue bar)

PBMC from healthy controls was examined in the same manner as for endothelial cells. Also here, hemozoin caused a dose-dependent release of IL-6 and as in HAoEC, rhIL-27 further enhanced the IL-6 release when co-incubated with hemozoin (50 μg/mL) (Fig. 4a-b). Moreover, hemozoin dose-dependently increased the release of IL-8, and as in HAoEC, rhIL-27 attenuated IL-8 release when co-incubated with hemozoin (200 μg/mL) (Fig. 4c-d). As in HAoEC, the maximal co-effect of rhIL-27 in hemozoin-exposed PBMC was observed at different concentrations of hemozoin depending on the actual cytokine (i.e., 50 μg/mL for IL-6 and 200 μg/mL for IL-8). The different concentrations in HAoEC as compared with PBMC suggest the sensitivity for the IL-27-mediated modulation of hemozoin-effects is not only dependent on the measured cytokine but also on cell type.

### Hemozoin upregulate IL-27Rα and gp130 expression in PBMC and HAoEC

**Fig. 5:**
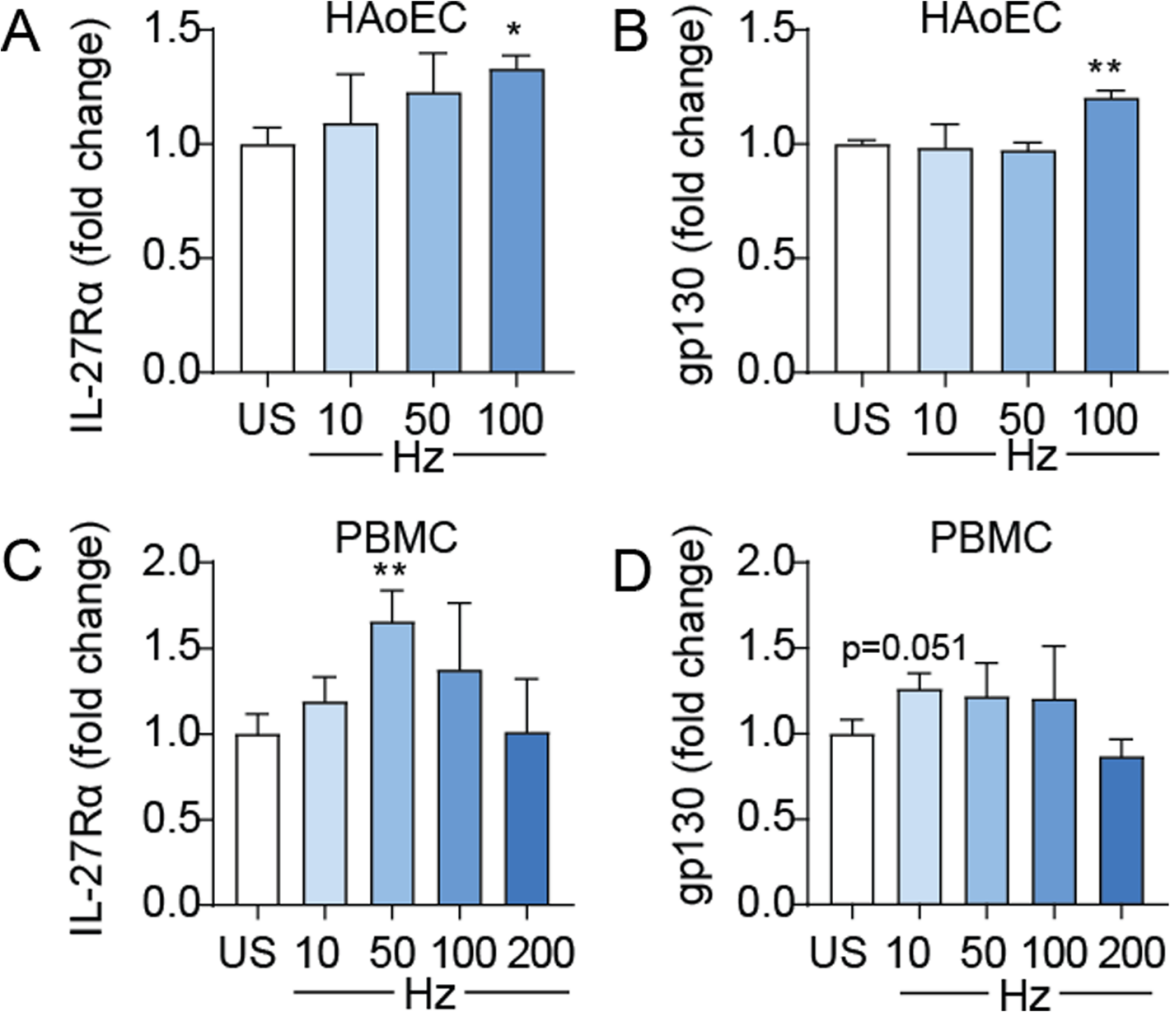
Effects of hemozoin on IL-27Rα and gp130 gene expression in HAoEC and PBMC. The cells were incubated with different concentrations of hemozoin (Hz) ranging from 10 to 200 μg/mL (indicated as Hz10, Hz50, Hz100 and Hz200) for five (**a**) and 22 (**b**-**d**) hours. Gene expression analyses were done by qPCR, related to reference gene β-actin/TaqMan reference probes and normalized to unstimulated cells (US). The figure shows mRNA levels of IL-27Rα and gp130 in HAoEC (**a** and **b**) and in PBMC (**c** and **d**). Results are representatives of minimum three experiments and data are presented as mean and SEM. *p < 0.05 and **p < 0.01 versus unstimulated cells (white bar)

Our findings show an interaction between hemozoin and IL-27 resulting in enhancing effects of IL-27 on hemozoin-induced IL-6 release and attenuating effect on IL-8 release. As shown in Fig. 5, hemozoin increased mRNA levels of both IL-27Rα and its co-receptor gp130 in PBMC and HAoEC. However, the effects were rather modest, and the effect on gp130 in PBMC was only borderline significant (*p* = 0.051).

## Discussion

Falciparum malaria is still a major challenge to the society in the developing countries and co-infection with HIV seems to worsen the disease course particular in pregnant women [23–25]. Here we show that plasma levels of IL-27 are markedly up-regulated in patients with falciparum malaria compared with HIV-infected patients with similar clinical symptoms but without malaria, and healthy controls, with no differences between those with and without co-infection with HIV. Moreover, whereas IL-27 levels were significantly correlated with *P. falciparum* parasitemia as assessed by qPCR in plasma and vWF as a marker of endothelial cell activation, we found no significant association with disease severity. Our in vitro experiments show that IL-27 modulated the hemozoin-mediated cytokine response in both endothelial cells and PBMC with enhancing effects on IL-6 and attenuating effects on IL-8. Our findings show that IL-27 is regulated during falciparum malaria in adults, potentially mediating both inflammatory and anti-inflammatory effects.

Decreased levels of IL-27 have been found in infants with severe falciparum malaria [26]. IL-27 levels are elevated in placental and cord blood compared with peripheral blood immediately following delivery in falciparum infected women [15], while no clear pattern was found during *P. vivax* malaria [27]. This is, however, the first report of IL-27 levels in adult patients with falciparum malaria demonstrating increased plasma levels as compared with healthy controls and HIV-infected patients with similar febrile illness, independent of co-infection with HIV. Interestingly, plasma IL-27 concentrations have been reported to be significantly decreased in untreated HIV-infected patients compared to healthy controls with a gradual increase after initiation of ART, potentially being involved in immune reconstitution following such therapy [28]. A larger study, however, found no change in plasma levels of IL-27 during HIV infection [29]. In addition, IL-27 levels seem to be increased during sepsis, and at least in children, potentially giving prognostic information in these patients [30, 31]. In this study, however, co-infection with other microbes such as those seen in the HIV-infected patients without malaria (e.g., tuberculosis, bacterial pneumonia and sepsis), did not seem to influence IL-27 levels to the same degree as co-infection with falciparum malaria. IL-27 appears mainly to be produced by antigen-presenting cells such as dendritic cells, macrophages and B cells. Interestingly, in a recent experimental study in mice infected with *P. berghei* ANKA, Kimura et al. identified a unique population of IL-27 producing regulatory CD4^+^ T cells [11]. Herein, we have no data on the cellular sources of IL-27 in human falciparum malaria, but notably, IL-27 levels were strongly correlated with plasma levels of IP-10 and sCD25 in patients with falciparum malaria, further suggesting a relation of IL-27 to T cell activation in malaria. However, these correlations were also seen in HIV-infected patients without falciparum malaria.

IL-27 has been demonstrated to possess both inflammatory (e.g., induction of Th1 related cytokines like interferon-γ) and anti-inflammatory (e.g., suppression of Th17 cells) responses [10], and more recently, IL-27 has been linked to enhanced IL-10 production in regulatory T cells [32]. Moreover, Kimura et al. have found that malaria-specific Foxp3^−^CD4^+^ T cells produced IL-27 and regulated IL-2 production and clonal expansion of effector CD4^+^ T cells during experimental malaria infection in mice [11]. In the present study we also, in our in vitro experiments, found both inflammatory and anti-inflammatory effects of IL-27. Thus, whereas IL-27 enhanced the spontaneous and hemozoin-induced release of IL-6, a cytokine related to IL-27, in both PBMC and endothelial cells, it attenuated IL-8 release in the same cells. The clinical relevance of these findings is unclear, but notably, we have shown markedly enhanced IL-8 levels in these patients with falciparum malaria, associated with disease severity and outcome [5]. Based on experimental studies, it has been suggested that IL-27, potentially induced by the parasite itself, could play a regulatory role in the maintenance of the balance between anti-malaria protective and host damaging immune responses [11, 33]. Our findings herein could potentially support such a notion by showing both inflammatory and anti-inflammatory responses of IL-27. Whereas the strong correlation of IL-27 with parasitemia could reflect enhancing effect on *P. falciparum* dissemination, it could also reflect a counteracting mechanism induced by the parasites. The reason for the lack of association of IL-27 levels with disease severity is at present not clear, but could in fact reflect the dual and regulatory properties of this cytokine, mediating both inflammatory and anti-inflammatory effects.

While endothelial cells seem to be a cellular source of IL-27 [34], only a few studies have examined the effects of IL-27 on these cells reporting both activating (i.e., enhanced TNF-mediated effects on adhesion molecules) and attenuating (i.e., inhibiting lymphatic endothelial cell proliferation) effects on cell activation [35, 36]. Herein we show both inflammatory (increased spontaneous and hemozoin-induced IL-6 release) and anti-inflammatory (attenuated spontaneous and hemozoin-induced IL-8 release). The strong correlation between IL-27 and vWF as a marker of endothelial cell activation also support a link between endothelial cells and IL-27 in vivo during falciparum malaria, either as a cellular source, cellular target or both.

There are several in vitro studies examining the interaction between hemozoin and different cell models showing at least in some degree different results. Several factors could have influenced these apparently discrepancies. Synthetic hemozoin has been shown to possess adjuvant properties that differ depending on the method of synthesis [37]. Native hemozoin can be purified from infected red blood cells in culture, and in order to obtain a pure product it needs to be further treated to remove any proteins, lipids, and other materials from disrupted parasites which may interfere with its stimulating profile. In contrast, synthetic hemozoin is totally free for parasite material, as for example malarial DNA, which has been shown to induce activation of Toll-like receptor 9 [38]. Synthetic hemozoin may have a larger crystal size than the native one, but the crystal size may differ dependent on the solvent used in the preparation procedure [37], and importantly, the crystal size will differently affect the production of inflammatory cytokines [37, 39, 40]. Further, sonicated hemozoin suspensions result in a stronger induction of cytokines than non-sonicated suspensions [37]. Herein, we used 10–200 μg/mL hemozoin which also has been used by others [41]. Lower concentrations has been suggested to be biological relevant [42], but it is not inconceivable that the hemozoin concentrations that were used in the present study could be found in clinical falciparum malaria at the site of inflammation with interactions between infected and ruptured erythrocytes and endothelial cells. Taken together, there are many factors that will affect the outcome of in vitro experiments, not only the use of synthetic or native hemozoin, but also in which way the hemozoin is synthesized, if sonication of hemozoin suspension is performed, the concentration of the crystals and also which cell model that is used. These issues must be taken into consideration in the interpretation of such in vitro data.

The present study has some limitations such as lack of clinical outcome data, and lack of in vitro experiments on cells obtained from the patients. Moreover, the lack of laboratory data on the control group as well as lack of CD4 T cell counts in the majority of the HIV-infected patients are also important limitations. The loss of malaria patients to follow-up at the 48-h time point, because of death, discharge or denial of second sampling, could have introduced confounding. Moreover, correlation data do not necessarily mean any causal relationship. Finally, we lack data that confirm similar in vitro data when using native hemozoin.

## Conclusions

Our data suggest that IL-27 is regulated during falciparum malaria independently of co-infection with HIV mediating both inflammatory and anti-inflammatory effects, potentially playing an immune-regulatory role during falciparum malaria. Our data may also support previous data from experimental studies on a regulatory role of IL-27 during malaria infection [11]. However, in relation to human malaria infection, this will have to be confirmed in larger clinical studies that also include studies on freshly isolated cells from the patient groups as well as data on clinical outcome.

## Data Availability

The datasets used and/or analyzed during the current study are available from the corresponding author on reasonable request.

## Abbreviations

EBI3: Epstein-Barr virus-induced gene 3
eGFR: estimated glomerular filtration rate
EIAs: Enzyme immunoassays
FBS: Fetal bovine serum
HAoECs: Primary Human Aortic Endothelial cells
IL: Interleukin
IP-10: Interferon gamma-induced protein 10
MCP-1: monocyte chemotactic protein-1
MDRD: “Modification of Diet in Renal Disease”, method for estimation of GFR
MIP-1β: Macrophage inflammatory protein-1β
*P. falciparum*: *Plasmodium falciparum*
PBMC: Peripheral Blood Mononuclear Cells
qPCR: real-time quantitative PCR
RDT: Rapid diagnostic test
rhIL-27: recombinant human IL-27
TNF: Tumor Necrosis Factor
vWF: von Willebrand factor
